# Supplementary figures and images for: Molecular insights into RNA recognition and gene regulation by the TRIM-NHL protein Mei-P26
Source: Life Sci Alliance. 2022 May 5;5(8):e202201418. doi: 10.26508/lsa.202201418 (PMC9070667; doi:10.26508/lsa.202201418)

Source Data: Figure 1

Figure 1 A

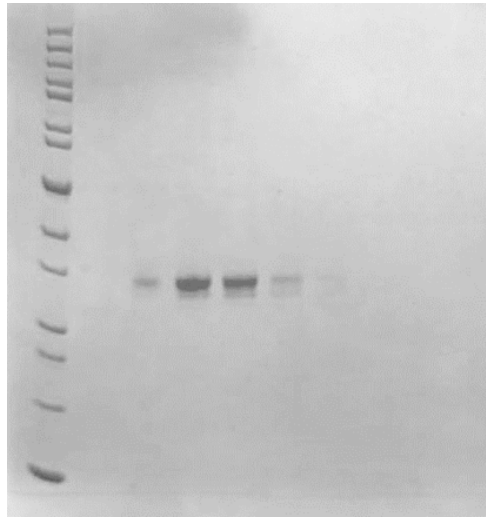

Figure 1 C

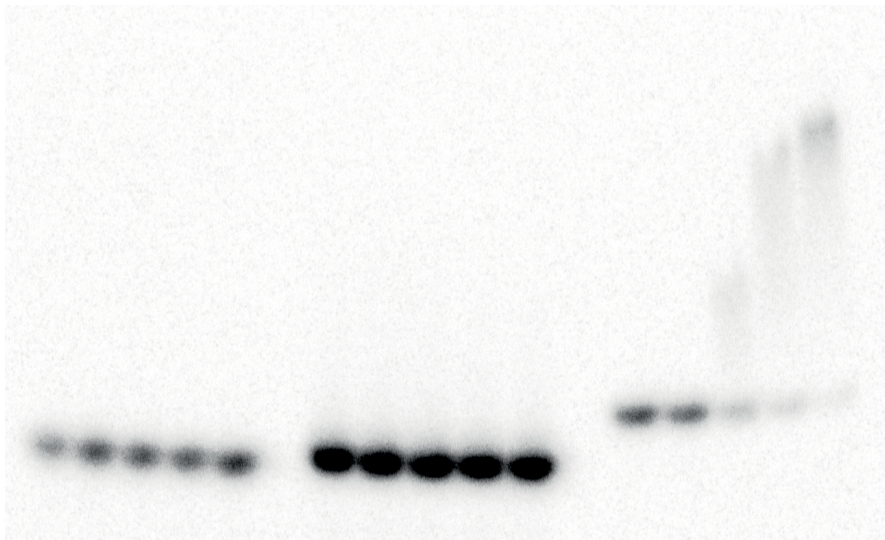

Supplement: Supplementary file 1 [file LSA-2022-01418_SdataF1.pdf]

Source Data: Supplementary Figure 1

Supplementary Figure 1 A

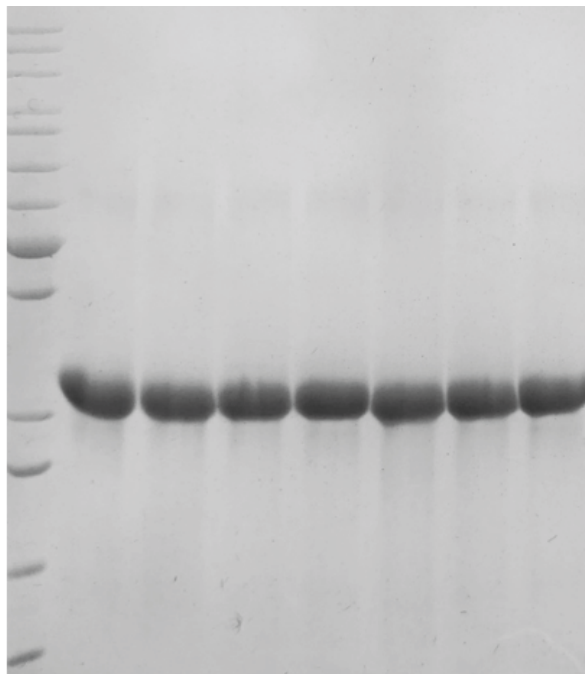

Supplementary Figure 1 B

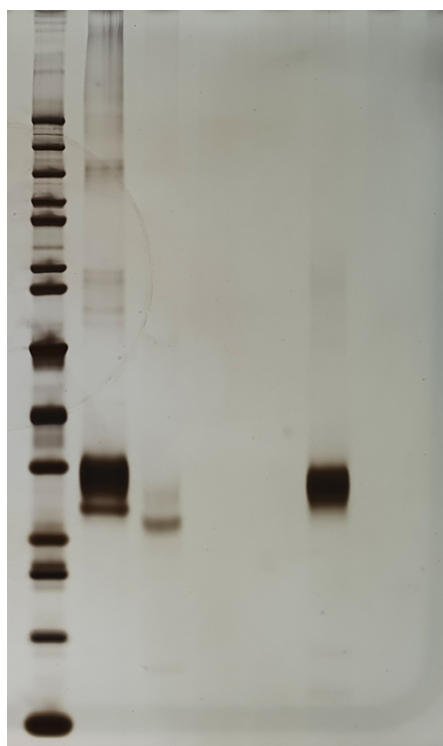

Supplement: Supplementary file 2 [file LSA-2022-01418_SdataFS1.pdf]

Source Data: Supplementary Figure 3

Supplementary Figure 3 A

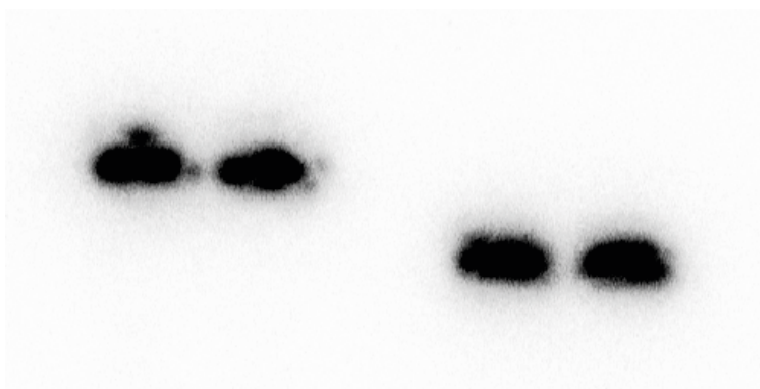

Supplementary Figure 3 C

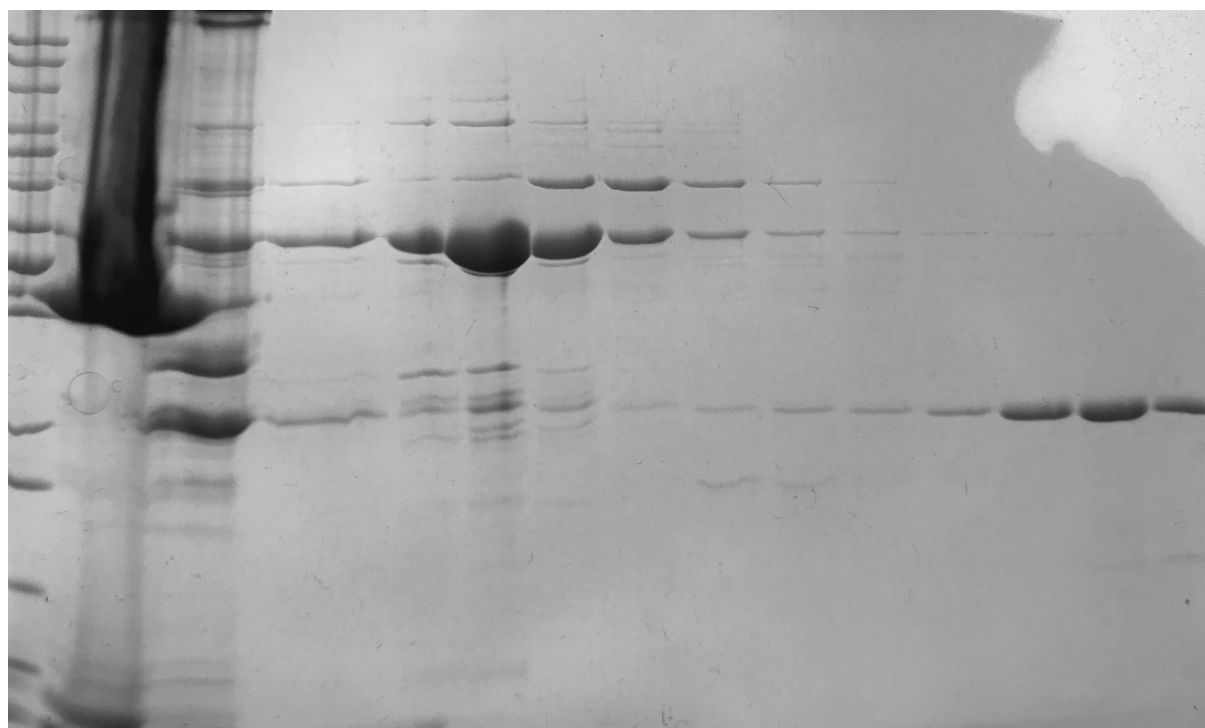

Supplement: Supplementary file 3 [file LSA-2022-01418_SdataFS3.pdf]

Source Data: Figure 3

Figure 3 B

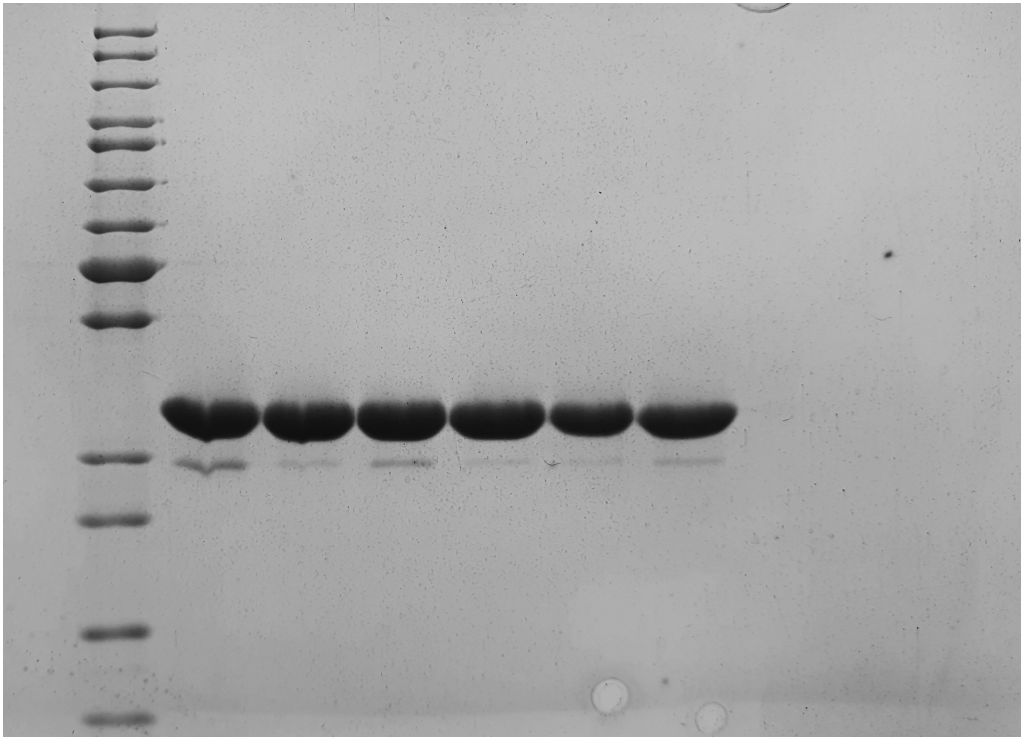

Figure 3 D

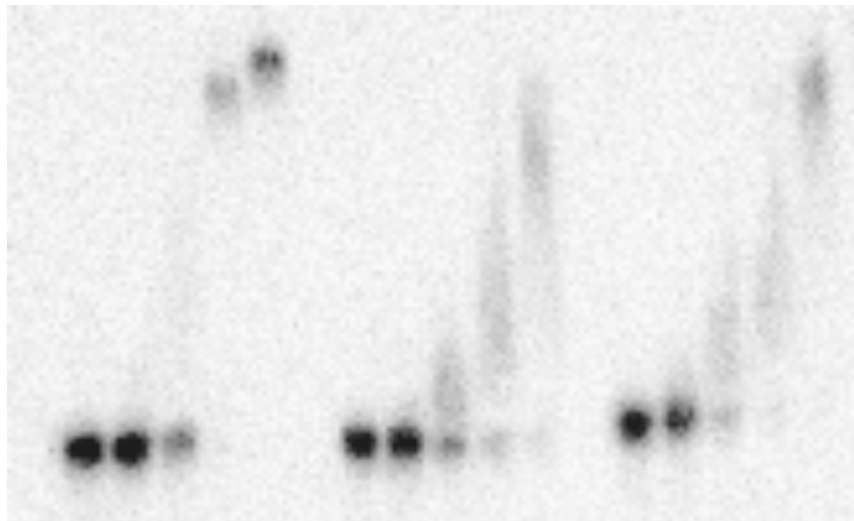

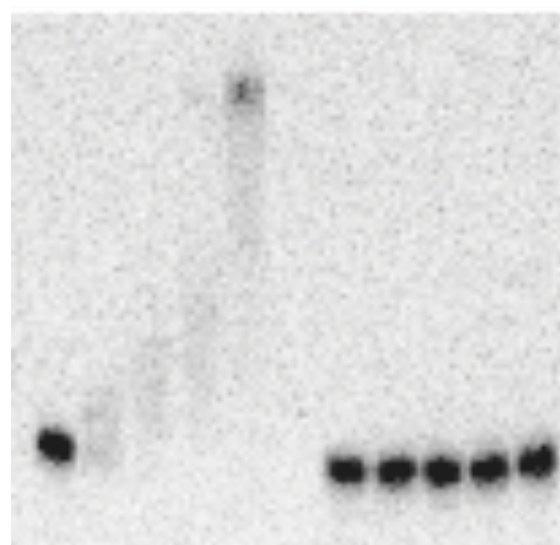

Supplement: Supplementary file 4 [file LSA-2022-01418_SdataF3.pdf]

Source Data: Supplementary Figure 7

Supplementary Figure 7 A

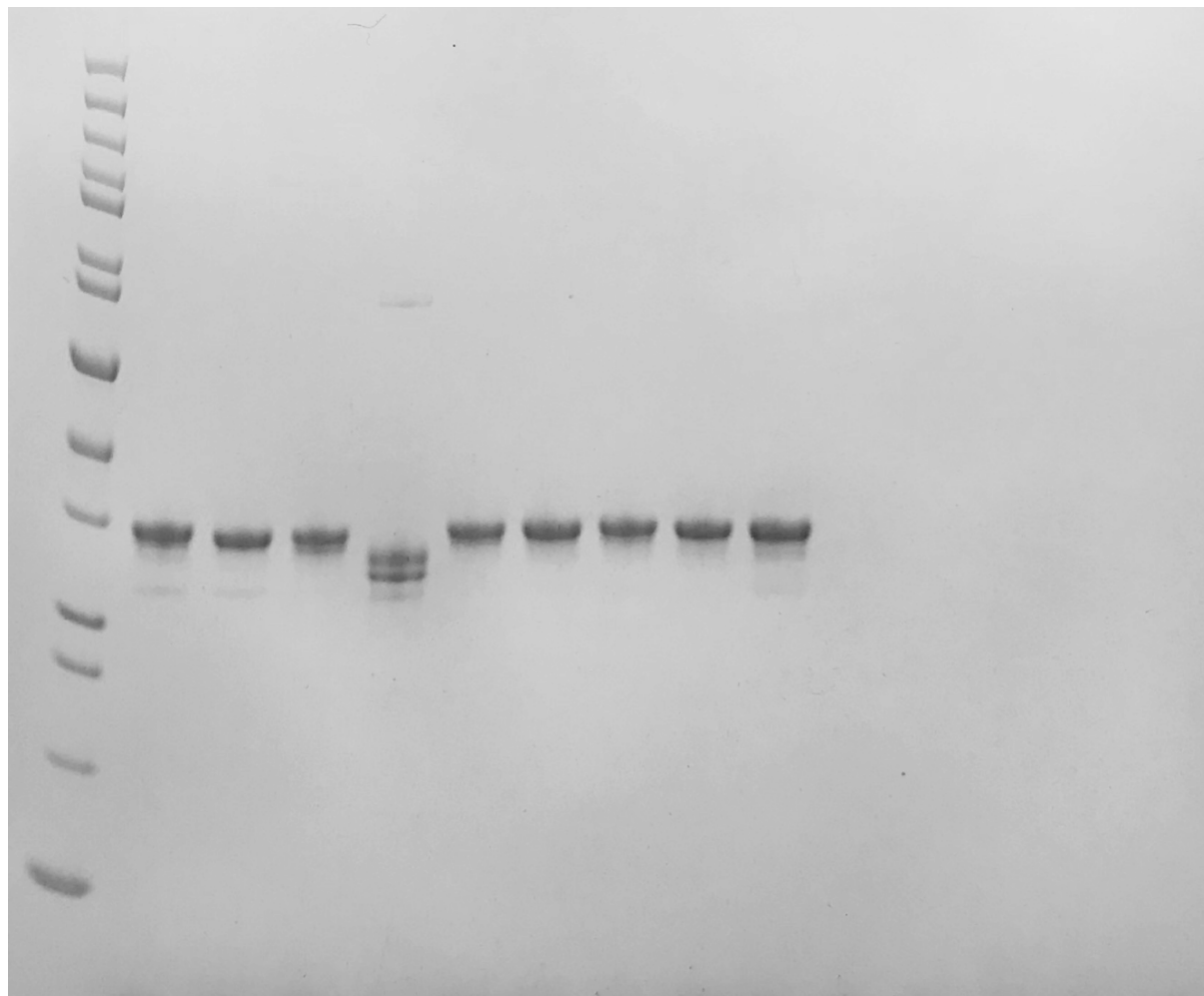

Supplement: Supplementary file 5 [file LSA-2022-01418_SdataFS7.pdf]

Source Data: Supplementary Figure 9

Supplementary Figure 9 A

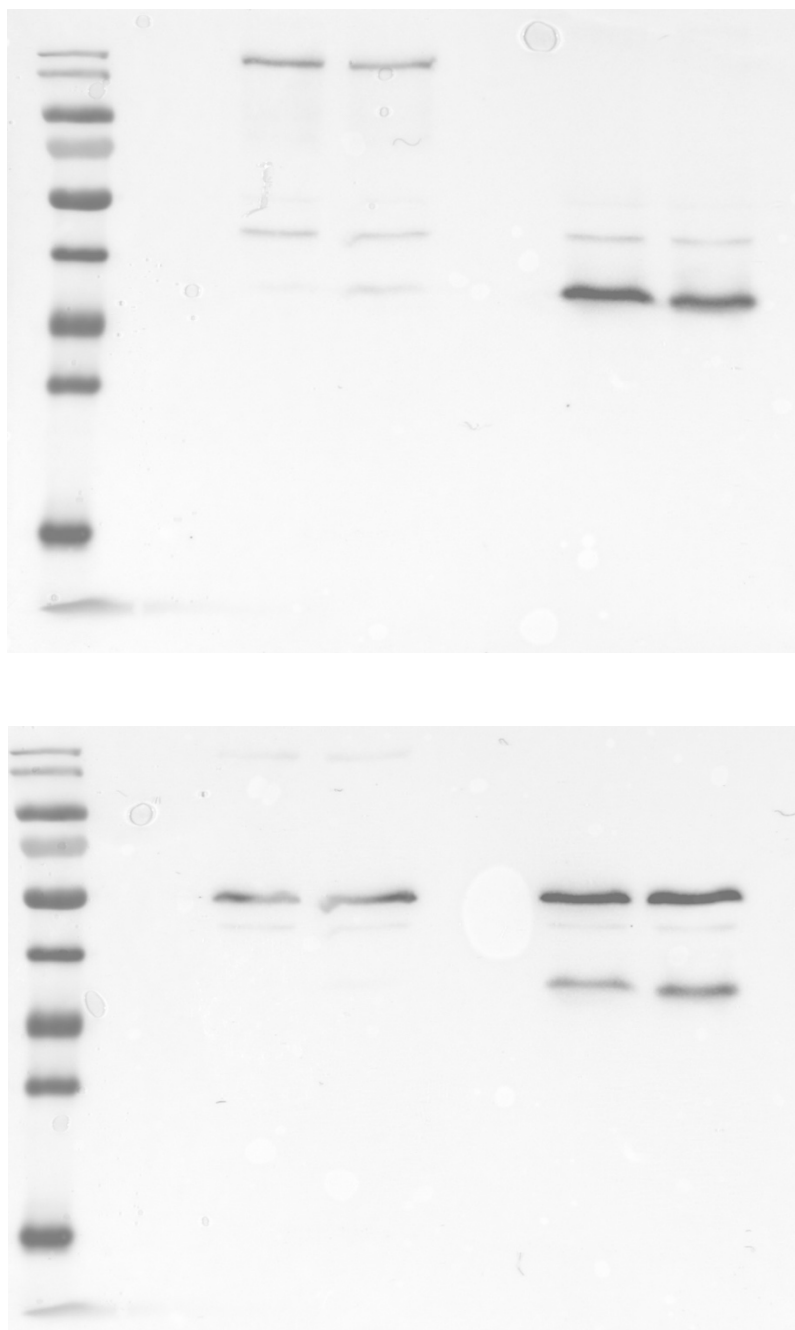

Supplementary Figure 9 B

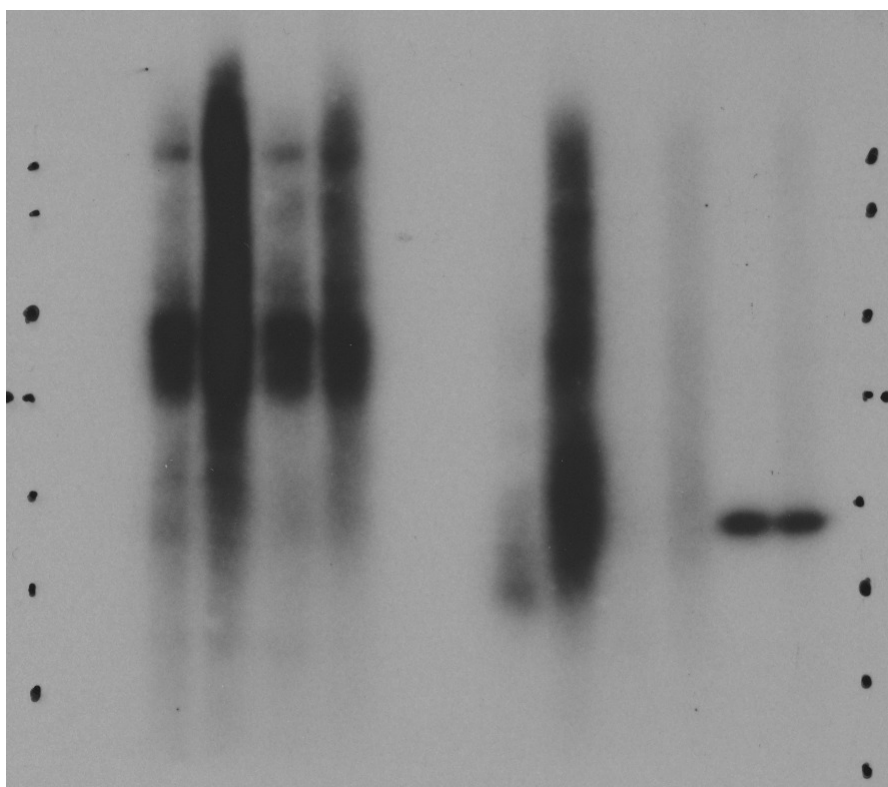

Supplement: Supplementary file 6 [file LSA-2022-01418_SdataFS9.pdf]

Source Data: Figure 4

Figure 4 F

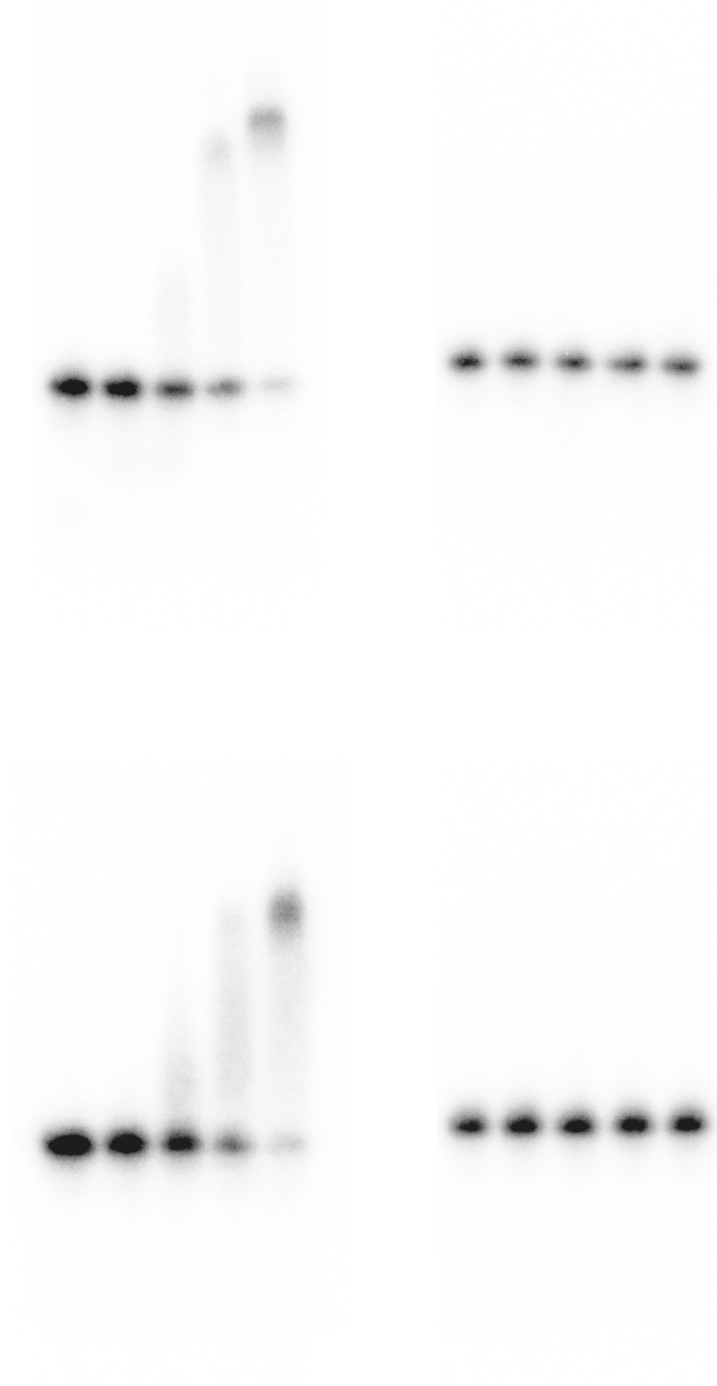

Supplement: Supplementary file 9 [file LSA-2022-01418_SdataF4.pdf]

Source Data: Supplementary Figure 11

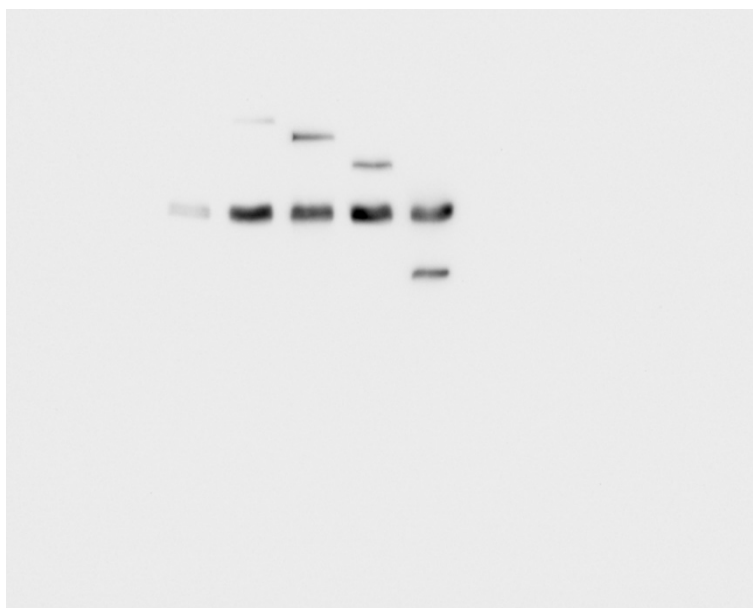

Supplement: Supplementary file 11 [file LSA-2022-01418_SdataFS11.pdf]
