# Supplementary material for: Molecular insights into RNA recognition and gene regulation by the TRIM-NHL protein Mei-P26
Source: Life Sci Alliance. 2022 May 5;5(8):e202201418. doi: 10.26508/lsa.202201418 (PMC9070667; doi:10.26508/lsa.202201418)

Source Data: Supplementary Figure 10

Supplementary Figure 10 A

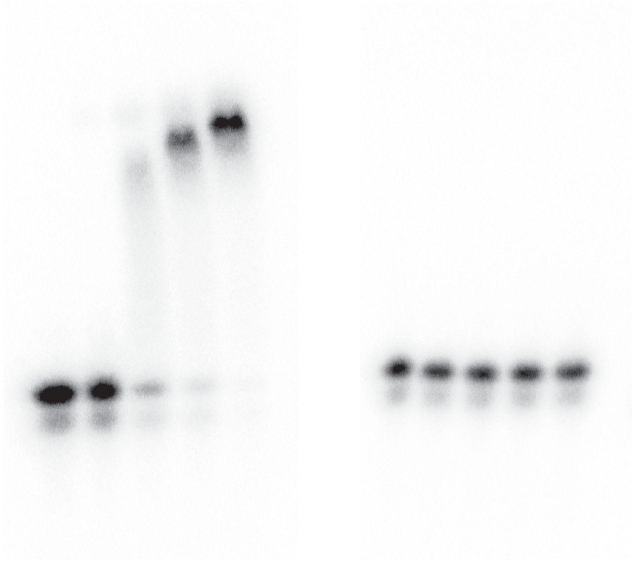

Supplementary Figure 10 B

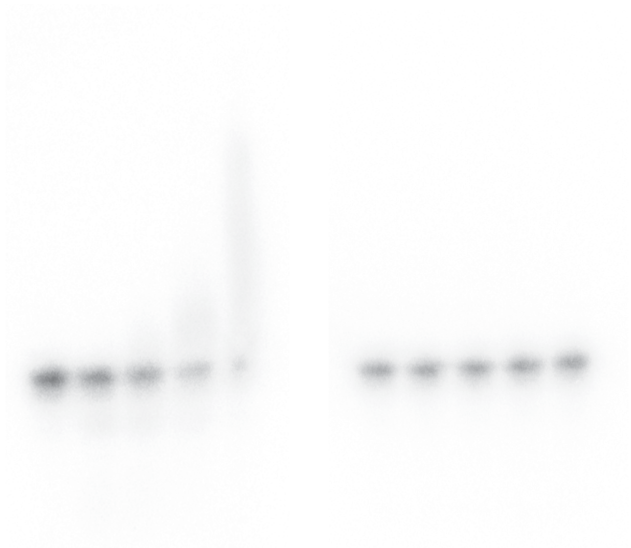

Supplementary Figure 10 C

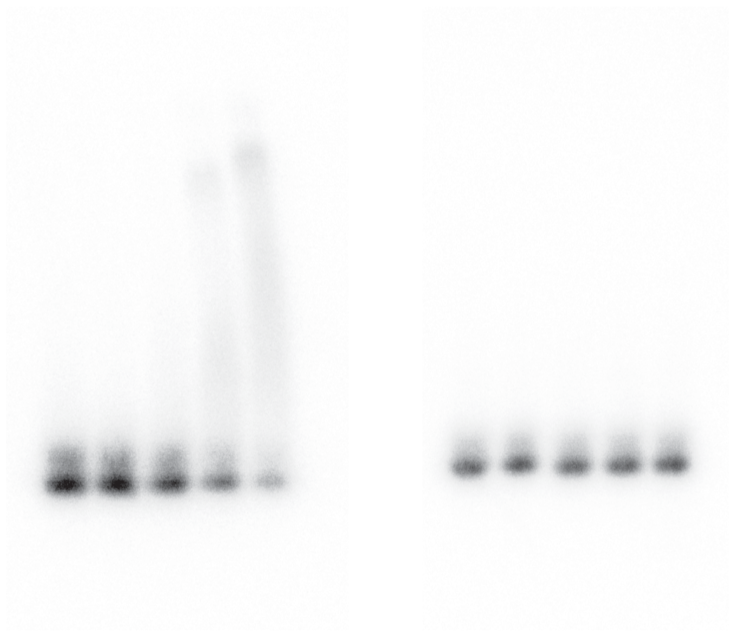

Supplement: Supplementary file 10 [file LSA-2022-01418_SdataFS10.pdf]
