# Supplementary material for: Molecular insights into RNA recognition and gene regulation by the TRIM-NHL protein Mei-P26
Source: Life Sci Alliance. 2022 May 5;5(8):e202201418. doi: 10.26508/lsa.202201418 (PMC9070667; doi:10.26508/lsa.202201418)
Supplement: Supplementary file 12 [file LSA-2022-01418_TableS1.docx]

## Supplementary Table 1. Used oligonucleotide sequences

| Name | Sequence 5′ - 3′ |
| --- | --- |
| Binding experiments |  |
| SEQ1 | UUUUUUU |
| SEQ2 | AUUUACA |
| SEQ3 | UUUUACA |
| SEQ4 | UUUACAA |
| SEQ5 | UUUUUAC |
| SEQ6 | UUUACAC |
| BRAT1 | UUGUUAA |
| BRAT_mut_ | UUUUUAA |
| dsRNA | GGAGUCCAACUCC |
| SEQ1_3G_ | UUGUUUU |
| SEQ1_4G_ | UUUGUUU |
| SEQ4_4U_ | UUUUACAA |
| SEQ3_5U_ | UUUUUACA |
| SEQ3_7U_ | UUUUUUUACA |
| SEQ3_Inv_ | ACAUUUU |
| SEQ3_CAC_ | UUUUCAC |
| DNA | TTTTACA |
| U_9_ | UUUUUUUUU |
| U_16_ | UUUUUUUUUUUUUUUU |
| Nos | UUAUAUAAUUUUUUUAUUUUUUUAAAAAAUGU |
| LanA | AUCCUUUUUAAAACUCC |
| Lost | CCCUUUUUUUAUACACAA |
| Mlc-C | GGUCUUUUUACAAAAG |
| Col4a1 | GCCAUUUUUUAUCUGCCA |
| Hrb27C | AGUAAUUUUUUCUAUUAAAGU |
|  |  |
| Cloning & mutagenesis |  |
| NHL_WT F | CCGTATCCTGCAGGCATGGGACTCAATAACTTC |
| NHL_WT R | CCGTATGCGGCCGCTCAGTGAACATAGAGGGTGTTCAG |
| K1172A_R1175A F | CGAGTGTCCCCATGTAGCGGTTTCCGCCTGCTGCGGACTAAAAATC |
| K1172A_R1175A R | GATTTTTAGTCCGCAGCAGGCGGAAACCGCTACATGGGGACACTCG |
| R1150A F | GGTGATTCGCACGGCAATGCCTTCCACGTCGCCTGC |
| R1150A R | GCAGGCGACGTGGAAGGCATTGCCGTGCGAATCACC |
| R1001A_K1002A F | GGCCAGCTCTGGTATCCGGCCGCGGTGGCCGTGATGCATAAC |
| R1001A_K1002A R | GTTATGCATCACGGCCACCGCGGCCGGATACCAGAGCTGGCC |
| K1192A F | CTATGTGGTTACGCTGGCAGCGAATAATCATCATGTTCTAGTCC |
| K1192A R | GGACTAGAACATGATGATTATTCGCTGCCAGCGTAACCACATAG |
| A1046R_F | ATTAGATACATTGATATCGTGCGTGGCTTGGCTGTCACAG |
| A1046R_R | CTGTGACAGCCAAGCCACGCACGATATCAATGTATCTAAT |
| dloop_P1169_R1175 F | CCTTCAGTCCGAGTTCGAGTGTGGTAGCGGCAGCGGTTGCTGCGGACTAAAAATCACATCCG |
| dloop_P1169_R1175 R | CGGATGTGATTTTTAGTCCGCAGCAACCGCTGCCGCTACCACACTCGAACTCGGACTGAAGG |
| R1017A_F | GGCAAATTTGTGGTCTGCGATGCTGGAAACGAACGTTCTCGC |
| R1017A_R | GCGAGAACGTTCGTTTCCAGCATCGCAGACCACAAATTTGCC |
| Y999A_F | GGAGGAGGGCCAGCTCTGGGCGCCGCGCAAGGTGGC |
| Y999A_R | GCCACCTTGCGCGGCGCCCAGAGCTGGCCCTCCTCC |
| K1172A_F | CGAGTGTCCCCATGTAGCGGTTTCCCGCTGCTGCGGACTAAAAATC |
| K1172A_R | GATTTTTAGTCCGCAGCAGCGGGAAACCGCTACATGGGGACACTCG |
| R1175A_F | CGAGTGTCCCCATGTAAAGGTTTCCGCCTGCTGCGGACTAAAAATC |
| R1175A_R | GATTTTTAGTCCGCAGCAGGCGGAAACCTTTACATGGGGACACTCG |
| 3ʹ UTR cloning |  |
| nos: nts 1654-2345 of CG5637-RA (FBtr0083732), the deletion encompasses nts 2247-2276 | |
| F nos | CGCTATGTTAACTATAGTATAGACAACGAACGATCACTCAAATC |
| R nos | CCGATAAGATCTTAAATTGTAACCATTTCTTTATTTGGCAC |
| F nos ΔBS | CACATGAAACAACCGCCAGCTGTGTACACATATTCTGAAAATGAAAAATTC |
| R nos ΔBS | GAATTTTTCATTTTCAGAATATGTGTACACAGCTGGCGGTTGTTTCATGTG |
| bic: nts 838-988 of bic-RB (FBtr0087783) | |
| bic F | CCGTATGTTAACATCGGAAACGGATCCACTC |
| bic R | CCGTATAGATCTGTTCTGTTCAGCGCTCACTC |
| chic: nts of 913-1120 of chic-RF (FBtr0309213) | |
| chic F | CCGTATGTTAACGTGTAGCAGAGGAAATGG |
| chic R | CCGTATAGATCTGCTGAGGACTGAATTCTTAAC |
| Col4a1: nts 5722-5930 of Col4a1-RB (FBtr0079002) | |
| Col4a1 F | CCGTATGTTAACCCAACACTGCTACAAATTCC |
| Col4a1 R | CCGTATAGATCTTTGGACATACGACCGAGGCTAG |
| eIF4A: nts 1551-1656 of eIF4A-RE (FBtr0307068) | |
| eIF4A F | CCGTATGTTAACCAGCAACACCACCACCAATTG |
| eIF4A R | CCGTATAGATCTTTTGTTGTTGCTGGTTGAC |
| Hrb27C: nts 3499-4135 of Hrb27C-RH (FBtr0307032) | |
| Hrb27C F | CCGTATGTTAACACTCTGCACGCTCACTAAAC |
| Hrb27C R | CCGTATAGATCTACAATCAGCCACGCACAC |
| Hsp83: nts 2686-2914 of Hsp83-RB (FBtr0332873) | |
| Hsp83 F | CCGTATGTTAACGCGTTATAAGCGACAGACATAC |
| Hsp83 R | CCGTATAGATCTATCATCGATCGGGTCATC |
| LanA: nts 11574-11650 of LanA-RA (FBtr0077014) | |
| LanA F | CCGTATGTTAACCTGCGTGCCAATTCTATTAAAGC |
| LanA R | CCGTATAGATCTCAAAAATGCAAGATTCTC |
| lost: nts 2387-2691 of lost-RA (FBtr0078921) | |
| lost F | CCGTATGTTAACGCCCTATAAATGAGAAATACCG |
| lost R | CCGTATAGATCTCGGTCAAGTTTATTTTTACG |
| Mlc-C: nts 1026-1248 of Mlc-c-RB (FBtr0308579) | |
| Mlc-C F | CCGTATGTTAACGGAAGGAAAGCCAGAGTATCAC |
| Mlc-C R | CCGTATAGATCTAAGGTTTCAATTGGTTTAATAG |
| RpS20: nts 454-552 of RpS20-RA (FBtr0083964) | |
| RpS20 F | CCGTATGTTAACGATCGGCAGATGCCACATTTTTAC |
| RpS20 R | CCGTATAGATCTTTCGCACAATAAAGTTGG |
| Rps23: nts 473-553 of RpS23-RB (FBtr0339970) | |
| Rps23 F | CCGTATGTTAACCTTAGATGAACATGCATCTAATC |
| RpS23 R | CCGTATAGATCTGTTTCTGTTTTTTGTTTATTTAG |
| spz: nts 1886-2053 of spt-RL (FBtr0114372) | |
| spz F | CCGTATGTTAACCTTCCACGTCTAATGCCTAAGC |
| spz R | CCGTATAGATCTGCTGGCATGTACTTGCATAC |
| sqd: nts 5364-5535 of sqd-RA (FBtr0082854) | |
| sqd F | CCGTATGTTAACGCCTTGCGCCATTTTCCTCG |
| sqd R | CCGTATAGATCTGCTCATTTTTCTTGTTTG |
| sta: nts 1022-1103 of sta-RA (FBtr0070289) | |
| sta F | CCGTATGTTAACAATCGTCCGGGCCACAGATG |
| sta R | CCGTATAGATCTAAACAGCTGTTTATTTATGC |
| Swip-1: nts 1680-1830 of Swip-1-RB (FBtr0340322) | |
| Swip-1 F | CCGTATGTTAACGCTTGGATCAGCCTAAAGTAAAAC |
| Swip-1 R | CCGTATAGATCTATTGCAGTATAAGTTTGTAG |
